# BEYOND THE CLINIC WALLS: HOW COMPREHENSIVE DEMENTIA CARE FOSTERS COMMUNITY ENGAGEMENT

**DOI:** 10.1093/geroni/igaf122.1415

**Published:** 2025-12-31

**Authors:** David Lee, Stacy Castellanos, Xi Zhu, Gery Ryan, Ron Hays, Luisa Blanco, David Reuben

**Affiliations:** University of California, Los Angeles David Geffen School of Medicine, Los Angeles, California, United States; University of California, Los Angeles Fielding School of Public Health, Los Angeles, California, United States; University of California, Los Angeles Fielding School of Public Health, Los Angeles, California, United States; Kaiser Permanente Southern California, Pasadena, California, United States; University of California, Los Angeles Department of Medicine, Division of General Internal Medicine & Health Services Research, Los Angeles, California, United States; Pepperdine School of Public Policy, Malibu, California, United States; University of California, Los Angeles, Los Angeles, California, United States

## Abstract

Comprehensive dementia care is a holistic approach to the complex medical and social needs of persons living with dementia and their caregivers. Six major evidence-based care models are being disseminated, each demonstrating psychosocial and healthcare-related benefits for patient and caregiver. This qualitative study explores how these models are delivered and create an environment that fosters community engagement. Semi-structured virtual interviews with at least two stakeholders (n = 44 interviewees) per organization (n = 22 organizations) across the six dementia care models were conducted, ranging from 29-58 minutes. Participants included managers (25%), care providers (43.2%), and individuals who held both roles (31.8%). Most organizations were healthcare systems (54.5%), while 45.5% were community-based. Qualitative codes were developed inductively and deductively, and thematic analysis was conducted. The following themes emerged indicating that some programs 1) Enhanced care coordination through structured community partnerships and having staff deliver services directly in the community (e.g., improving access to resources like meal delivery services and adult day services, using community-health workers who did home visits) (community embeddedness); 2) Used virtual communication methods to expand service coverage, enabling more frequent and broader community engagement (community expansion); 3) Created social support and educational opportunities for patients and caregivers to provide emotional support, foster caregiving self-efficacy, and promote community among other patients and caregivers (e.g., support programs, dementia education programs/virtual calls, memory cafes) (community creation). These findings highlight how comprehensive dementia care programs foster care coordination, enhance accessibility, and provide essential social and educational support for patients and caregivers.

